# Impact of Inflammatory Cytokines on Effector and Memory CD8+ T Cells

**DOI:** 10.3389/fimmu.2014.00295

**Published:** 2014-06-19

**Authors:** Marie T. Kim, John T. Harty

**Affiliations:** ^1^Interdisciplinary Program in Immunology, University of Iowa, Iowa City, IA, USA; ^2^Department of Microbiology, University of Iowa, Iowa City, IA, USA; ^3^Department of Pathology, University of Iowa, Iowa City, IA, USA

**Keywords:** signal 3, cytokines, effector, resident memory, memory, CD8 T cells

## Abstract

Inflammatory cytokines have long been recognized to produce potent APCs to elicit robust T cell responses for protective immunity. The impact of inflammatory cytokine signaling directly on T cells, however, has only recently been appreciated. Although much remains to be learned, the CD8 T cell field has made considerable strides in understanding the effects of inflammatory cytokines throughout the CD8 T cell response. Key findings first identified IL-12 and type I interferons as “signal 3” cytokines, emphasizing their importance in generating optimal CD8 T cell responses. Separate investigations revealed another inflammatory cytokine, IL-15, to play a critical role in memory CD8 T cell maintenance. These early studies highlighted potential regulators of CD8 T cells, but were unable to provide mechanistic insight into how these inflammatory cytokines enhanced CD8 T cell-mediated immunity. Here, we describe the mechanistic advances that have been made in our lab regarding the role of “signal 3” cytokines and IL-15 in optimizing effector and memory CD8 T cell number and function. Furthermore, we assess initial progress on the role of cytokines, such as TGF-β, in generation of recently described resident memory CD8 T cell populations.

## Introduction

Naïve CD8 T cells undergo activation when presented with their cognate antigen following a three-signal model. Professional antigen-presenting cells (APCs) provide the crucial first and second signals through the T cell receptor (TCR) and costimulatory molecules, while innate immune cells contribute inflammatory cytokines to promote optimal accumulation and differentiation of effector CD8 T cells (1). Although the role of inflammatory cytokines in maturing professional APCs to stimulate robust T cell responses has been well described (2), investigation of their direct effect on T cells is ongoing. In the following review, we outline mechanistic studies identified for inflammatory cytokine regulation of various stages of the CD8 T cell response and discuss cutting edge research on the cytokine requirements for generation of the novel resident memory T cell (T_RM_) population.

## Signal 3 Cytokines and the Magnitude of the Effector CD8 T Cell Response

Initial studies suggesting that cytokines, particularly IL-12, may be important for signaling directly to T cells were made in *in vitro* cultures of T cells and artificial APCs more than a decade ago. Since then, the importance of IL-12 and type I interferon signaling directly to CD8 T cells for optimal effector cell accumulation has been demonstrated both *in vitro* and *in vivo* (3–7). Despite the clear impact of IL-12 and IFNα/β on effector CD8 T cell numbers, it remained unclear how inflammatory cytokines regulated the magnitude of effector CD8 T cell responses. Although several other cytokines have been discussed in the literature recently for T cell differentiation (8, 9); here, we will focus on signal 3 cytokines as originally defined for their role in T cell accumulation (3–7). After their classification as signal 3 cytokines, IL-12 and type I interferons were proposed to enhance accumulation of CD8 T cells following one of two models: via greater survival (2, 10) or by conferring an early proliferative advantage (11, 12). The model for enhanced survival stemmed from 3 days culture experiments, which demonstrated accumulations of cells in cultures containing IL-12 with no detectable changes in cell division. The latter model was supported also by *in vitro* studies, where IL-12 transiently increased expression of CD25, the high affinity IL-2 receptor, peaking at day 2 (11). Hence, previous reports addressing the mechanism by which signal 3 cytokines allow optimal accumulation of effector CD8 T cells were limited to short-term *in vitro* experiments with no clear answer to the question of whether survival or early proliferation, or both, contribute to the magnitude of the CD8 T cell response. Furthermore, the temporal disconnect between signal 3 cytokine-driven CD25 expression and optimal accumulation of effector CD8 T cells many days later has not been assessed (13). Here, we describe a recent study from our lab addressing these knowledge gaps concerning the mechanism by which signal 3 cytokines allow optimal accumulation of effector CD8 T cells *in vivo*.

Utilizing an OT-I T cell adoptive transfer system followed by DC-OVA priming with or without the TLR9 agonist, CpG, to induce signal 3 cytokines, Starbeck-Miller et al. compared CD8 T cells activated *in vivo* in the presence or absence of signal 3 cytokines (14). Gene expression profiling of T cells from these groups at D7 post immunization clearly showed that signal 3 cytokines enhanced transcription of proliferation, but not anti-apoptosis-associated genes (14). Additionally, analysis of CD8 T cells primed by DC with or without signal 3 showed no differences in proliferation or total cells numbers as late as day 5 post immunization. Thus, the *in vivo* data do not support either of the proposed models for signal 3 activity. Interestingly, both DC and DC + CpG OT-I cells isolated on D4 and moved into *in vitro* cultures failed to divide, although transfer of the same populations to an *in vivo* host revealed more robust proliferation from the CD8 T cells that had been exposed to signal 3 cytokines. This suggested that signal 3 cytokines established a proliferation program, but sustained proliferation required an additional component that was present in a naïve host. Since IL-2 is an important driver of T cell accumulation, Starbeck-Miller et al. monitored expression of the high affinity IL-2 receptor, CD25, on DC versus DC + CpG CD8 T cells. Indeed, IL-12 and type I interferon sustained CD25 expression, allowing for greater IL-2-induced proliferation via activation of the PI3K pathway and expression of FoxM1, a positive cell cycle gene regulator. Importantly, administering the IL-2 neutralizing antibody JES6 from D4-6 removed the proliferative advantage conferred by signal 3 cytokines. Thus, these studies verify, and add mechanistic insight to the model, indicating that signal 3 cytokines neither enhance survival not provide and early proliferative advantage, but rather sustain expression of the high affinity IL-2 receptor, which extends the duration of proliferation after immunization and permits optimal generation of effector CD8 T cells *in vivo*. Interestingly, the effects of IL-12 and type I interferons are not limited to promoting optimal CD8 T cell accumulation, but offer functional advantages to effector CD8 T cells, such as antigen sensitivity, which will be discussed next.

## Dynamic Regulation of Antigen Sensitivity by Inflammatory Cytokines

The protective capacity of CD8 T cells depends on their quantity, functional properties, and anatomical distribution (15). High antigen sensitivity, otherwise referred to as functional avidity, strongly correlates with protective immunity against intracellular pathogens (16). Although T cells cannot directly alter the binding affinity of their TCR through processes like somatic hypermutation, it has been shown that monoclonal TCR-transgenic CD8 T cells can increase their functional avidity from early to late effector time points (17). This study suggested that the functional avidity maturation was a fixed property of CD8 T cells. Here, we describe a mechanistic study demonstrating that inflammatory cytokines directly enhance antigen sensitivity of effector and memory CD8 T cells, however this enhanced sensitivity is not hardwired, but rather tuned by the pathogen-specific milieu.

Using a similar DC immunization protocol as indicated previously, Richer et al. activated OT-I CD8 T cells in the presence or absence of signal 3 cytokines (18). Distinct inflammatory milieu were then initiated by co-infection of DC primed mice with *Listeria monocytogenes* (Lm) or lymphocytic choriomeningitis virus (LCMV) and antigen sensitivity was assessed at day 5 after priming. Strikingly, DC-OVA with LCMV infection substantially enhanced antigen sensitivity by more than 10-fold whereas co-infection with Lm enhanced antigen-sensitivity four to sixfold. To determine whether inflammation increased functional avidity via enhanced TCR signaling, Richer et al. isolated OT-I T cells from DC and DC + LCMV mice on D4 and analyzed phosphorylation of downstream TCR signals after TCR ligation (18). Indeed, inflammatory cytokines dramatically enhanced phosphorylation of ZAP-70, PLCgamma, and ERK1/2 in response to TCR stimulation. Importantly, greater ERK1/2 phosphorylation was not observed with PMA stimulation, which bypasses proximal TCR signals, suggesting that inflammatory cytokines increased the antigen sensitivity of the TCR by enhancing proximal TCR signaling. Consistent with the data from effector CD8 T cells, inflammatory cytokines also increased the antigen sensitivity of memory CD8 T cells by enhancing TCR proximal signaling, albeit to a lesser degree than observed with effector CD8 T cells. This study demonstrated how the pathogen-specific inflammatory milieu affects antigen-sensitivity, an essential functional aspect of both effector and memory CD8 T cells. In addition to signal 3 cytokine effects on memory CD8 T cells, we next review a novel role for IL-15 in memory CD8 T cell trafficking.

## IL-15-Dependent Synthesis of Selectin Ligands

Numerous studies have described the functional differences between memory and naïve CD8 T cells (13, 19). Among such reports, it was demonstrated that memory, but not naïve, CD8 T cells can be rapidly recruited to inflamed lungs in an antigen-independent manner (20). Importantly, this large influx of memory CD8 T cells was shown to provide immediate cytolytic killing against pathogens expressing cognate antigen (21). Although this non-specific recruitment of memory CD8 T cells was shown to depend on CCR5 expression, the molecular mechanisms initiating early “tethering and rolling” events before chemokine recognition by memory CD8 T cells detection remained undefined.

Immune cell homing is a highly regulated process that begins with selectin family proteins. Leukocytes extravasate into inflamed tissue by constructing ligands to P- and E-selectin, which are expressed on activated endothelium. In contrast, L-selectin mediates homeostatic trafficking of naïve and central memory CD8 T cells through lymph nodes. Previous reports concerning the synthesis of P- and E-selectin ligands had been limited to *in vitro* models, which suggested TCR activation was essential to express appropriate selectin ligands. Herein, we describe studies from Nolz et al. that show P- and E-selectin ligand synthesis occurs on memory, but not naïve, CD8 T cells following inflammation *in vivo* (22). Utilizing the model pathogen, LCMV, Nolz et al. observed uniform expression of functional P- and E-selectin ligands on effector populations, but that most memory CD8 T cells did not express functional P or E-selectin ligands. After detecting high selectin ligand expression on non-specifically recruited memory P14 CD8 T cells following several irrelevant pathogen infections, it was demonstrated, through use of blocking antibodies to P- and E-selectin or P-selectin glycoprotein ligand-1, that non-specific recruitment of memory CD8 T cells to inflamed sites was dependent on selectin binding. To investigate the mechanism regulating inflammation-induced selectin ligand expression on memory CD8 T cells, Nolz et al. analyzed expression of the *Gcnt1* gene, which prompts their formation on naïve, effector, and memory CD8 T cells. Although effector CD8 T cells expressed high levels of *Gcnt1*, naïve, and memory CD8 T cells had minimal expression of this protein. Interestingly, recombinant IL-15 substantially enhanced P- and E-selectin ligand synthesis on memory, but not naive CD8 T cells *in vitro* and Nolz et al. revealed a similar induction of the Gcnt1 protein via immunoblot. *In vivo*, IL-15-deficiency significantly reduced expression of selectin ligands, and subsequent memory CD8 T cell trafficking to inflamed sites, suggesting that P- and E-selectin ligand expression occurs in an IL-15/STAT5-dependent, but TCR-independent manner. Importantly, IL-15-driven P- and E-selectin ligand expression was shown to occur in human memory CD8 T cells, demonstrating conserved trafficking pathways between mouse and human T cells that can be manipulated for therapeutic purposes.

Until now, IL-15 has been referred to, principally, as a maintenance cytokine for memory CD8 T cells. This study investigating the role of IL-15 in the regulation of core 2 O-glycan synthesis on memory CD8 T cells suggests the possibility of other unexplored functions of this important inflammatory cytokine.

## TGF-β, IL-33, and TNF Required for Resident Memory CD8 T Cells

Although the CD8 T cell field has established a paradigm of IL-15-driven homeostatic proliferation as the model of memory CD8 T cell maintenance for circulating T cells, localized CD8 T cell populations in the lung (23), gut (24), and skin (25), among other tissues, have been shown to sustain a sizable pool of memory CD8 T cells despite the absence of IL-15 signaling. Most recently, the T_RM_ population has garnered immense interest for their distinct surface phenotype, local protective capacity, and long-term maintenance in the absence of traditional cytokines. Skin and gut infection models to generate transgenic CD8+ T_RM_ populations are well established (26, 27); hence, we describe recent advances in determining the cytokine signals involved for T_RM_ development and maintenance following either immunization or infection.

Resident memory T cell cells represent a novel, non-circulating class of T cells that persist within extralymphoid tissue and demonstrate superior regional immunity (28). The best-characterized T_RM_ cells express the alpha chain of the αEβ7 integrin (CD103), as well as the sphingosine 1 phosphate receptor (S1PR_1_) inhibitor CD69, in multiple tissue compartments. Relevantly, both molecules are required for the optimal formation and maintenance of T_RM_ cells in the skin (26). Since *in vitro* and some *in vivo* studies have long since shown that transforming growth factor-β (TGF-β) signaling promotes CD103 expression on immune cells (29–31) and that TGF-β is expressed in the skin epithelium, Mackay et al. investigated whether signaling through the TGF-β receptor was required to upregulate CD103 and establish T_RM_ cells *in vivo* (26, 32). Utilizing one to one adoptive transfer models of WT and *Tgfbr2^f/f^*.dLck-Cre (Tgfbr2−/−) OT-I T cells into C57BL/6 mice followed by infection with OVA-expressing HSV, Mackay et al. indeed demonstrated that Tgfbr2−/− OT-I cells failed to upregulate CD103 and had a dramatically reduced ability to form T_RM_.

By utilizing acute and chronic infections with LCMV, Zhang et al. delved further into the mechanism behind TGF-β signaling for generation and maintenance of T_RM_ cells (27). Creating equal ratio mixtures of WT and Tgfbr2−/− P14 T cells followed by either LCMV-Armstrong (acute) or Clone 13 (Cl13, chronic) infections, Zhang et al. notices defective maintenance of Tgfbr2−/− cells in Armstrong, but not Cl13-infected hosts. While monitoring integrin expression in secondary lymphoid organs, which are the major source for T_RM_ cells, Zhang et al. detected enhanced expression of α4β7 on Tgfbr2−/− cells in Cl13, compared to Armstrong-infected mice (27). As α4β7 aids in the migration to the gut (33, 34), it was concluded that, although Tgfbr2−/− T_RM_ cells are locally declining in both Armstrong and Cl13-infected mice, the more prominent, enhanced expression of α4β7 on splenic Tgfbr2−/− T cells of Cl13-infected hosts allowed for continual replacement and stabilization of T_RM_ numbers. Thus, TGF-β acts as a negative regulator to T_RM_ formation through α4β7 downregulation, but is required for the maintenance of established T_RM_ cells in the gut through induction of CD103 expression.

**Figure 1:**
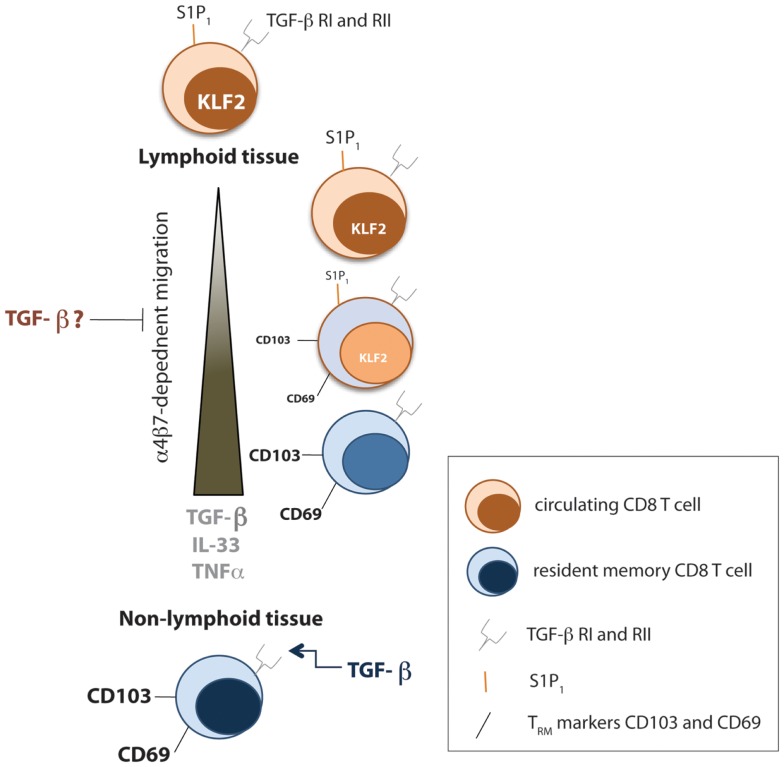
**Cytokines involved in TRM formation and maintenance**. Early after infection, local TGF-β signals prevent migration of effector CD8 T cells from the spleen to non-lymphoid tissue by downregulating the αEb7 integrin. However, tissue-specific programming during priming of CD8 T cells causes homing to appropriate resident tissue. In addition, the transcription factor KLF2 gets downregulated as effector CD8 T cells travel to non-lymphoid tissue toward a combination of TGF-β, IL-33, and TNFα signals, which causes a decrease in S1P1, allowing upregulation of CD69. Within resident tissue, TGF-β signals sustain TRM numbers.

The above findings clearly identify the relationship between TGF-β and CD103 expression for persistence of T_RM_ cells; however, CD103 is not required in all T_RM_ niches (35–37). Thus, we outline a complementary study, defining the transcriptional regulation of a ubiquitous T_RM_ marker, CD69, to establish T_RM_ cells, where CD103 may be dispensable. The antagonistic relationship between CD69 and S1PR_1_ are well established (38). The zinc-finger transcription factor KLF2 catalyzes the expression of S1PR_1_, known to promote lymph node egress (39). Hence, Skon et al. initially uses adoptive transfer models of KLF2-GFP P14 T cells followed by LCMV-Armstrong infection to monitor KLF2 expression in circulating, compared to resident memory CD8 T cells (40). As expected, T_RM_ cells expressed low levels of both KLF2 and S1PR_1_, while CD69 expression was increased. Interestingly, *in vitro* cytokine screening revealed that a combination of TGF-β, IL-33, and TNF were capable of inducing a modest downregulation of KLF2 expression. To analyze the effect of S1PR_1_ expression on T_RM_ formation, Skon et al. overexpressed S1PR_1_ through retroviral transduction of P14 cells, and demonstrated that failure to downregulate S1PR_1_ prevented the establishment of T_RM_ cells in the salivary gland, kidney, lamina propria, and intestinal epithelium (40). Hence, these studies propose that migration to non-lymphoid tissue enhances exposure of CD8 T cells to TGF-β, IL-33, and TNF, which triggers some loss of KLF2 expression, subsequently decreasing S1PR_1_, and allowing CD69 upregulation. Although the upregulation of CD69 may be controlled by multiple factors, increasing the complexity of this process, these studies, among others, clearly demonstrate that the non-migratory T_RM_ population has novel cytokine requirements for their generation and maintenance (Figure 1) and that this list of cytokines may continue to expand.

## Synopsis

In this review, we outline recent studies uncovering the mechanisms by which inflammatory cytokines regulate various attributes of circulating and resident memory CD8 T cell populations. Although investigation of the role of inflammatory cytokines on T_RM_ cells, and T cells in general, remain far from complete, the field has made remarkable progress in understanding how the inflammatory environment can directly modulate the number, function, migration, and maintenance of T cells.

## Conflict of Interest Statement

The authors declare that the research was conducted in the absence of any commercial or financial relationships that could be construed as a potential conflict of interest.

## References

[1] Haring JS Badovinac VP Harty JT Inflaming the CD8+ T cell response. Immunity (2006) 25(1):19–29 10.1016/j.immuni.2006.07.001 16860754

[2] Mitchell TC Hildeman D Kedl RM Teague TK Schaefer BC White J Immunological adjuvants promote activated T cell survival via induction of Bcl-3. Nat Immunol (2001) 2(5):397–402 11323692 10.1038/87692

[3] Curtsinger JM Mescher MF Inflammatory cytokines as a third signal for T cell activation. Curr Opin Immunol (2010) 22(3):333–40 10.1016/j.coi.2010.02.013 20363604 PMC2891062

[4] Curtsinger JM Schmidt CS Mondino A Lins DC Kedl RM Jenkins MK Inflammatory cytokines provide a third signal for activation of naive CD4+ and CD8+ T cells. J Immunol (1999) 162(6):3256–62 10092777

[5] Gately MK Wolitzky AG Quinn PM Chizzonite R Regulation of human cytolytic lymphocyte responses by interleukin-12. Cell Immunol (1992) 143(1):127–42 10.1016/0008-8749(92)90011-D 1352483

[6] Trinchieri G Interleukin-12: a cytokine at the interface of inflammation and immunity. Adv Immunol (1998) 70:83–243 10.1016/S0065-2776(08)60387-9 9755338

[7] Xiao Z Casey KA Jameson SC Curtsinger JM Mescher MF Programming for CD8 T cell memory development requires IL-12 or type I IFN. J Immunol (2009) 182(5):2786–94 10.4049/jimmunol.0803484 19234173 PMC2648124

[8] Cui W Liu Y Weinstein JS Craft J Kaech SM An interleukin-21-interleukin-10-STAT3 pathway is critical for functional maturation of memory CD8+ T cells. Immunity (2011) 35(5):792–805 10.1016/j.immuni.2011.09.017 22118527 PMC3431922

[9] Siegel AM Heimall J Freeman AF Hsu AP Brittain E Brenchley JM A critical role for STAT3 transcription factor signaling in the development and maintenance of human T cell memory. Immunity (2011) 35(5):806–18 10.1016/j.immuni.2011.09.016 22118528 PMC3228524

[10] Valenzuela JO Hammerbeck CD Mescher MF Cutting edge: Bcl-3 up-regulation by signal 3 cytokine (IL-12) prolongs survival of antigen-activated CD8 T cells. J Immunol (2005) 174(2):600–4 10.4049/jimmunol.174.2.600 15634875

[11] Valenzuela J Schmidt C Mescher M The roles of IL-12 in providing a third signal for clonal expansion of naive CD8 T cells. J Immunol (2002) 169(12):6842–9 10.4049/jimmunol.169.12.6842 12471116

[12] Curtsinger JM Valenzuela JO Agarwal P Lins D Mescher MF Type I IFNs provide a third signal to CD8 T cells to stimulate clonal expansion and differentiation. J Immunol (2005) 174(8):4465–9 10.4049/jimmunol.174.8.4465 15814665

[13] Harty JT Badovinac VP Shaping and reshaping CD8+ T-cell memory. Nat Rev Immunol. (2008) 8(2):107–19 10.1038/nri2251 18219309

[14] Starbeck-Miller GR Xue HH Harty JT IL-12 and type I interferon prolong the division of activated CD8 T cells by maintaining high-affinity IL-2 signaling in vivo. J Exp Med (2013) 211(1):105–20 10.1084/jem.20130901 24367005 PMC3892973

[15] Zhang N Bevan MJ CD8(+) T cells: foot soldiers of the immune system. Immunity (2011) 35(2):161–8 10.1016/j.immuni.2011.07.010 21867926 PMC3303224

[16] Alexander-Miller MA High-avidity CD8+ T cells: optimal soldiers in the war against viruses and tumors. Immunol Res (2005) 31(1):13–24 10.1385/IR:31:1:13 15591619

[17] Slifka MK Whitton JL Functional avidity maturation of CD8(+) T cells without selection of higher affinity TCR. Nat Immunol (2001) 2(8):711–7 10.1038/90650 11477407

[18] Richer MJ Nolz JC Harty JT Pathogen-specific inflammatory milieux tune the antigen sensitivity of CD8(+) T cells by enhancing T cell receptor signaling. Immunity (2013) 38(1):140–52 10.1016/j.immuni.2012.09.017 23260194 PMC3557574

[19] Nolz JC Starbeck-Miller GR Harty JT Naive, effector and memory CD8 T-cell trafficking: parallels and distinctions. Immunotherapy. (2011) 3(10):1223–33 10.2217/imt.11.100 21995573 PMC3214994

[20] Ely KH Cauley LS Roberts AD Brennan JW Cookenham T Woodland DL Nonspecific recruitment of memory CD8+ T cells to the lung airways during respiratory virus infections. J Immunol (2003) 170(3):1423–9 10.4049/jimmunol.170.3.1423 12538703

[21] Gebhardt T Whitney PG Zaid A Mackay LK Brooks AG Heath WR Different patterns of peripheral migration by memory CD4+ and CD8+ T cells. Nature (2011) 477(7363):216–9 10.1038/nature10339 21841802

[22] Nolz JC Harty JT IL-15 regulates memory CD8+ T cell O-glycan synthesis and affects trafficking. J Clin Invest (2014) 124(3):1013–26 10.1172/JCI72039 24509081 PMC3934158

[23] Verbist KC Field MB Klonowski KD Cutting edge: IL-15-independent maintenance of mucosally generated memory CD8 T cells. J Immunol (2011) 186(12):6667–71 10.4049/jimmunol.1004022 21572025 PMC3110618

[24] Masopust D Vezys V Wherry EJ Barber DL Ahmed R Cutting edge: gut microenvironment promotes differentiation of a unique memory CD8 T cell population. J Immunol (2006) 176(4):2079–83 10.4049/jimmunol.176.4.2079 16455963

[25] Jiang X Clark RA Liu L Wagers AJ Fuhlbrigge RC Kupper TS Skin infection generates non-migratory memory CD8+ T(RM) cells providing global skin immunity. Nature (2012) 483(7388):227–31 10.1038/nature10851 22388819 PMC3437663

[26] Mackay LK Rahimpour A Ma JZ Collins N Stock AT Hafon ML The developmental pathway for CD103(+)CD8+ tissue-resident memory T cells of skin. Nat Immunol (2013) 14(12):1294–301 10.1038/ni.2744 24162776

[27] Zhang N Bevan MJ Transforming growth factor-beta signaling controls the formation and maintenance of gut-resident memory T cells by regulating migration and retention. Immunity (2013) 39(4):687–96 10.1016/j.immuni.2013.08.019 24076049 PMC3805703

[28] Shin H Iwasaki A Tissue-resident memory T cells. Immunol Rev (2013) 255(1):165–81 10.1111/imr.12087 23947354 PMC3748618

[29] Wang D Yuan R Feng Y El-Asady R Farber DL Gress RE Regulation of CD103 expression by CD8+ T cells responding to renal allografts. J Immunol (2004) 172(1):214–21 10.4049/jimmunol.172.1.214 14688328

[30] Keskin DB Allan DS Rybalov B Andzelm MM Stern JN Kopcow HD TGFbeta promotes conversion of CD16+ peripheral blood NK cells into CD16- NK cells with similarities to decidual NK cells. Proc Natl Acad Sci USA (2007) 104(9):3378–83 10.1073/pnas.0611098104 17360654 PMC1805591

[31] Coombes JL Siddiqui KR Arancibia-Carcamo CV Hall J Sun CM Belkaid Y A functionally specialized population of mucosal CD103+ DCs induces Foxp3+ regulatory T cells via a TGF-beta and retinoic acid-dependent mechanism. J Exp Med (2007) 204(8):1757–64 10.1084/jem.20070590 17620361 PMC2118683

[32] El-Asady R Yuan R Liu K Wang D Gress RE Lucas PJ TGF-{beta}-dependent CD103 expression by CD8(+) T cells promotes selective destruction of the host intestinal epithelium during graft-versus-host disease. J Exp Med (2005) 201(10):1647–57 10.1084/jem.20041044 15897278 PMC2212926

[33] Bargatze RF Jutila MA Butcher EC Distinct roles of L-selectin and integrins alpha 4 beta 7 and LFA-1 in lymphocyte homing to Peyer’s patch-HEV in situ: the multistep model confirmed and refined. Immunity (1995) 3(1):99–108 10.1016/1074-7613(95)90162-0 7542550

[34] Hamann A Andrew DP Jablonski-Westrich D Holzmann B Butcher EC Role of alpha 4-integrins in lymphocyte homing to mucosal tissues in vivo. J Immunol (1994) 152(7):3282–93 7511642

[35] Hofmann M Pircher H E-cadherin promotes accumulation of a unique memory CD8 T-cell population in murine salivary glands. Proc Natl Acad Sci USA (2011) 108(40):16741–6 10.1073/pnas.1107200108 21930933 PMC3189029

[36] Casey KA Fraser KA Schenkel JM Moran A Abt MC Beura LK Antigen-independent differentiation and maintenance of effector-like resident memory T cells in tissues. J Immunol (2012) 188(10):4866–75 10.4049/jimmunol.1200402 22504644 PMC3345065

[37] Ariotti S Haanen JB Schumacher TN Behavior and function of tissue-resident memory T cells. Adv Immunol (2012) 114:203–16 10.1016/B978-0-12-396548-6.00008-1 22449783

[38] Bankovich AJ Shiow LR Cyster JG CD69 suppresses sphingosine 1-phosophate receptor-1 (S1P1) function through interaction with membrane helix 4. J Biol Chem (2010) 285(29):22328–37 10.1074/jbc.M110.123299 20463015 PMC2903414

[39] Matloubian M Lo CG Cinamon G Lesneski MJ Xu Y Brinkmann V Lymphocyte egress from thymus and peripheral lymphoid organs is dependent on S1P receptor 1. Nature (2004) 427(6972):355–60 10.1038/nature02284 14737169

[40] Skon CN Lee JY Anderson KG Masopust D Hogquist KA Jameson SC Transcriptional downregulation of S1pr1 is required for the establishment of resident memory CD8+ T cells. Nat Immunol (2013) 14(12):1285–93 10.1038/ni.2745 24162775 PMC3844557

